# Advances in hypothalamic hamartoma research over the past 30 years (1992–2021): a bibliometric analysis

**DOI:** 10.3389/fneur.2023.1176459

**Published:** 2023-06-06

**Authors:** Di Lu, Tianren Wang, Yanfeng Yang, Xiaotong Fan, Sichang Chen, Penghu Wei, Yongzhi Shan, Guoguang Zhao

**Affiliations:** ^1^Department of Neurosurgery, Xuanwu Hospital, Capital Medical University, Beijing, China; ^2^Clinical Research Center for Epilepsy, Capital Medical University, Beijing, China; ^3^Beijing Municipal Geriatric Medical Research Center, Beijing, China

**Keywords:** hypothalamic hamartoma, bibliometrics, gelastic seizures, precocious puberty, MRg-LITT = MR-guided laser interstitial thermal therapy, RF-TC = radiofrequency thermocoagulation

## Abstract

**Background:**

Hypothalamic hamartoma (HH) is a rare intracranial disease whose manifestations include gelastic seizures and precocious puberty. The diagnosis and treatment of HH have changed substantially over the past three decades as medical care has improved. Bibliometrics can reveal the evolution and development of a scientific field.

**Methods:**

Documents on HH were retrieved from the Web of Science Core Collection (WoSCC) database on September 8, 2022. The search terms were as follows: “hypothalamic hamartoma” or “hamartoma of the hypothalamus” or “hypothalamic hamartomas.” The types of documents were restricted to articles, case reports, and reviews. VOSviewer, CiteSpace, and the R package “bibliometrix” were used for a bibliometric analysis.

**Results:**

A total of 667 independent documents on HH were obtained from the WoSCC database. The most common types of documents were articles (*n* = 498, 75%) and reviews (*n* = 103, 15%). The number of annual publications fluctuated but showed an upward trend overall, and the annual growth rate was 6.85%. The cumulative publication data indicated that the most influential journals in the HH field include *Epilepsia*, *Epileptic Disorders*, *Child’s Nervous System*, *Neurosurgery*, and the *Journal of Neurosurgery*. Kerrigan JF, Ng YT, Rekate HL, Regis J, and Kameyama S were among the most prominent authors in the field of HH, with numerous publications and citations. American research institutions, especially the Barrow Neurological Institute, occupied a pivotal position in HH research. Other countries and institutions were catching up and producing considerable research results. Research on HH has steadily switched its emphasis from Pallister-Hall syndrome (PHS) and precocious puberty to epilepsy and new diagnostic and therapeutic techniques, including Gamma Knife, laser ablation, and interstitial thermal therapy.

**Conclusion:**

HH remains a special neurological disease with significant research prospects. The development of novel technologies, including MRI-guided laser-induced thermal therapy (MRg-LiTT) and stereotactic radiofrequency thermocoagulation (RF-TC), has enabled the efficient treatment of gelastic seizures in HH while minimizing the risks associated with craniotomies. Through bibliometric analysis, this study points out the direction for future HH research.

## 1. Introduction

Hypothalamic hamartoma (HH) is a rare intracranial disease entity with an incidence rate of approximately 2 per 100,000. It is a nonneoplastic lesion that develops from the tuber cinereum and inferior hypothalamus (1). On the basis of its radiographic appearance, HH can be broadly organized into three categories: intrahypothalamic, mixed hypothalamic, and parahypothalamic (2). Despite its generally benign histopathological nature, a range of symptoms can be present in individuals due to the specific anatomic sites. Gelastic seizures are the most distinctive clinical manifestations of HH. They present with bursts of laughter concomitant with generalized tonic–clonic or atonic seizures that are difficult to treat with antiepileptic drugs (3). In addition, HH may lead to precocious puberty (4). Psychiatric comorbidities and cognitive impairments are also common phenomena in HH patients (5).

Therefore, medical intervention is required for HH. Previously, only symptomatic therapies were accessible, including antiepileptic drugs (6) and hormonal therapy (7). Later, with the continuous development of neurosurgery, surgical treatment became the main treatment modality (8). However, hypothalamic operations can present risks, such as hypernatremia and hyponatremia (9). In addition to craniotomy, Gamma Knife surgery has also exhibited a certain degree of efficacy for refractory epilepsy caused by HH (10). Recently, HH therapy has reached a new stage with the growth of the epileptic network theory and minimally invasive techniques such as stereoelectroencephalography (SEEG), radiofrequency thermocoagulation (RF-TC) (11, 12), and magnetic resonance imaging (MRI)-guided laser interstitial thermal therapy (MRg-LiTT) (13–15). Many institutions, including Xuanwu Hospital of Capital Medical University, have conducted in-depth research on HH (1, 16–18). The change in treatment modalities for HH exemplifies the continuous and dramatic developments in epilepsy neurosurgery. Therefore, it is imperative to gain a more in-depth understanding through scientific inquiry.

Bibliometric analysis is a well-established and rigorous mathematical method for the analysis of large amounts of scientific literature. It can guide future clinical and research work in HH by providing a quantitative understanding of scientific trends and citation patterns. It can also identify emerging areas of interest and research gaps to dictate research agendas and funding priorities. By identifying influential authors and institutions, it can facilitate the collaboration and mentoring. Additionally, bibliometric analysis can be used to track the dissemination of new therapeutic theories, as well as to assess the effectiveness of treatments designed to improve the prognosis. To date, a specific bibliometric analysis of HH research has not yet been performed. Through bibliometric analyses, we delved deeper into the evolution and development of the study of HH and surgical intervention for intractable epilepsy.

The present study was guided by the following research questions:

1. What are the historical developmental pathways of HH research?
2. What progress has been made to date in HH research?
3. What are the research gaps and the potentially fruitful directions for future research in HH?
4. Who played a pivotal role in the study of HH? Who are the potential mentoring experts and collaborators?
5. What are the important journals, institutions, and countries in the field of HH research?

## 2. Materials and methods

### 2.1. Document retrieval and search strategy

**Figure 1:**
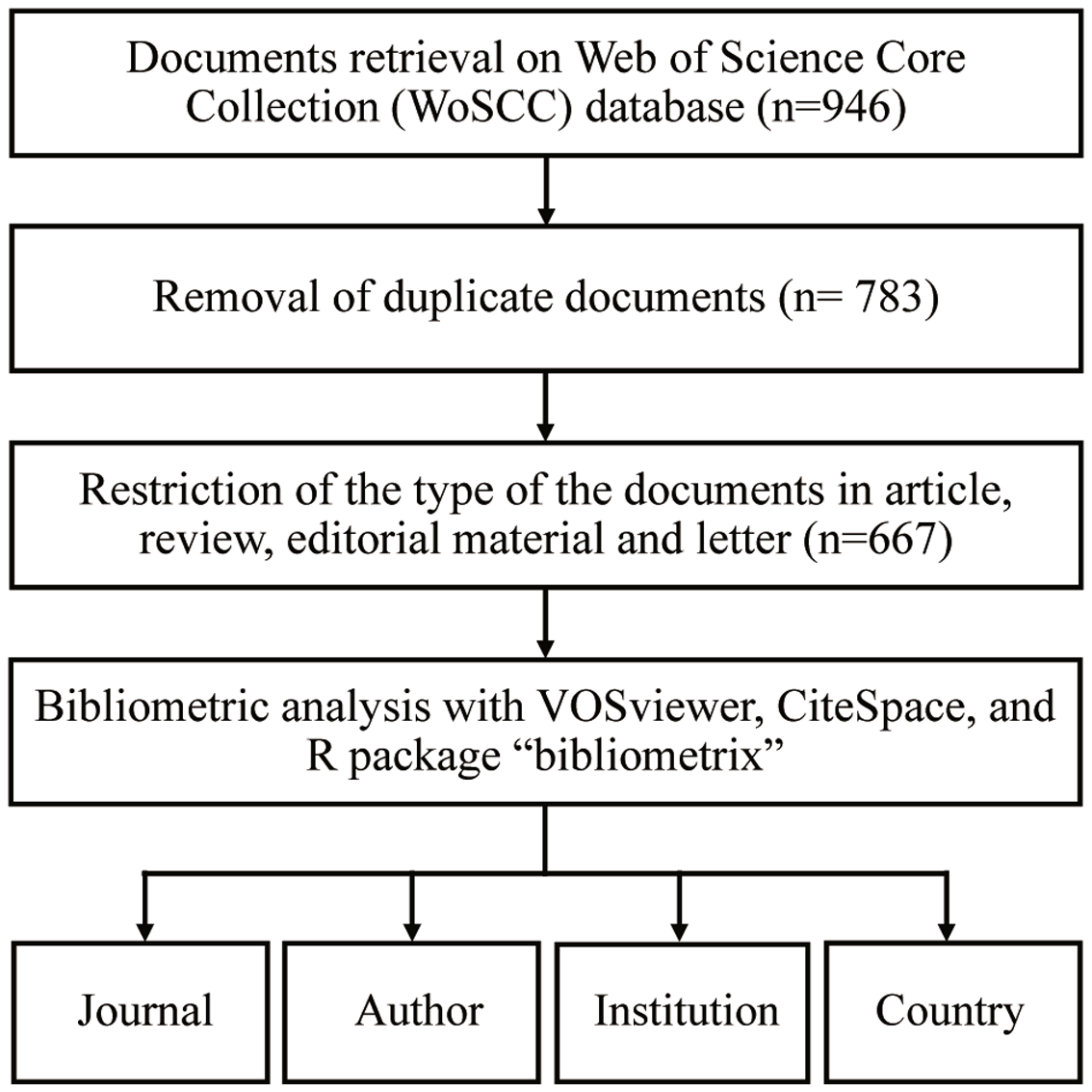
Flowchart of the study.

We performed a comprehensive and in-depth search of the Web of Science Core Collection (WoSCC). The edition was Science Citation Index Expanded (SCI-EXPANDED). According to the Medical Subject Headings (MeSH) terminology of the National Library of Medicine,^1^ the following search terms were used: TS = (“hypothalamic hamartoma”) or TS = (“hamartoma of the hypothalamus”) or TS = (“hypothalamic hamartomas”). The types of documents were restricted to articles, case reports, and reviews; documents published from 1992 to 2021 were considered, and no language restrictions were imposed. The results were exported, with full records and cited references, to two independent plain text files, as the maximum number of records in each exported file was 500. Each file contained a variety of basic information of the literature, including the authors’ names (AU and AF), titles (TI), publication name (SO), language (LA), document type (DT), abstracts (AB), keywords (DE), and cited references (CR), etc. Then, we renamed these two files “download_.txt,” enabling CiteSpace software to recognize and analyze them. We retrieved and downloaded the results on the same day (September 8, 2022) to avoid any potential bias or discrepancy due to the daily database update. Articles that did not meet the research objectives and those that were duplicates of included studies were removed by two investigators independently. Any disagreement was resolved by a third investigator. A flowchart of the retrieval process is shown in Figure 1.

### 2.2. Data analysis

We used various platforms, including VOSviewer, CiteSpace, and the R package “bibliometrix,” for the bibliometric analysis.

VOSviewer (version 1.6.18) is a fast and free bibliometric software program created by Nees Jan van Eck and Professor Ludo Waltman at Leiden University (19). This software was used for co-authorship, co-occurrence, and co-citation analysis. Furthermore, we performed a thorough analysis of the retrieved files from three perspectives, namely, authors, organizations, and countries, with network, overlay, and density visualization.

CiteSpace software (version 6.1. R3 basic) was developed by Professor Chaomei Chen for scientometric analysis and visualization (20). It was implemented in a Java environment. CiteSpace has powerful analytic capabilities, which can be used to calculate node centrality, link strength, cluster exploration, citation bursts, etc. Additionally, various parameters in the control panel allow the user to customize the visualization results, including the colormap, burstiness graph, clusters, labels, layout, and views.

The R package “bibliometrix” was first released in 2016 by Massimo Aria and Corrado Cuccurullo (21). In the present study, “biblioshiny,” a web interface for bibliometrix, was applied to RStudio version 1.2.5042. This application was used to implement several unique bibliometric analysis methods, including analyses of the conceptual, intellectual, and social structures of knowledge. It also provided some special perspectives through visualization, such as word clouds, thematic maps, and topic dendrograms. The website also provided a variety of parameters for rigorous quantitative evaluation, including the H-index and G-index. The H-index, also known as the Hirsch index, is defined as the highest value for which an author has at least H documents cited at least H times. The G-index refers to the value for which the top G documents have received at least G^2^ citations when documents are ranked in decreasing order based on the number of citations (22).

In addition, this study used Excel software (version 2019) and linear regression analysis for statistics and prediction of the publications over the years.

## 3. Results

### 3.1. Analysis of publications

**Figure 2:**
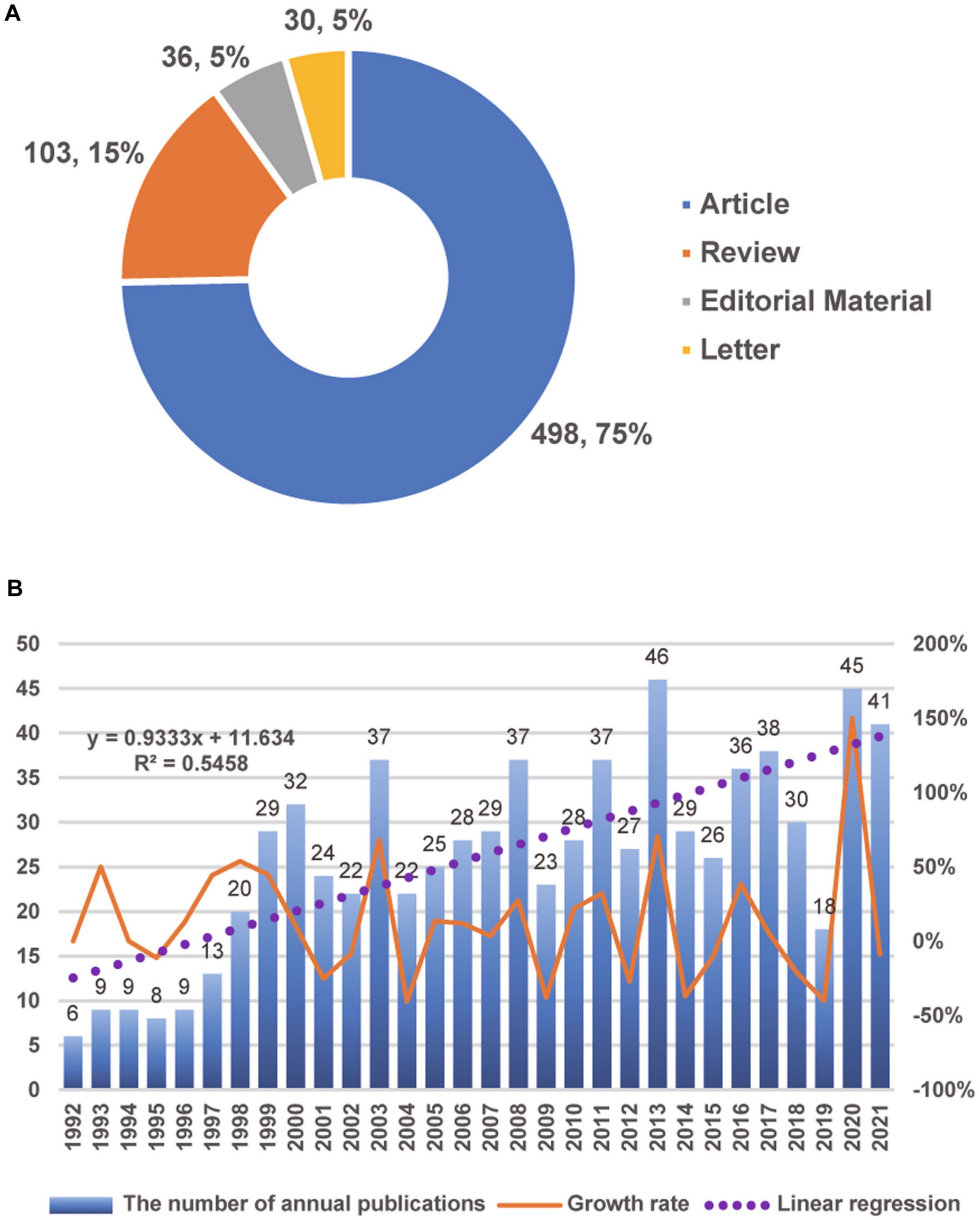
Analysis of the publications. **(A)** The type and proportion of HH documents with independent DOI numbers. **(B)** The annual number of publications and the growth rate from 1992 to 2021.

To some extent, the number of publications on a given topic in journals is an indication of the research trends and popularity of the field. In addition, it can shed light on the progress of diagnosis and therapeutic strategies. In this study, a total of 946 documents on HH from the past three decades were obtained from the WoSCC database. CiteSpace was used to eliminate duplicate literature (one document), corrections (three), meeting abstracts (150), and notes (9). The number of remaining documents with unique records by source was 783 (Supplementary Table S1). Furthermore, 667 (85.2%) of these publications included a complete and independent Digital Object Identifier (DOI) number. The most common types of documents were articles (*n* = 498; 75%) and reviews (*n* = 103; 15%) (Figure 2A).

The number of annual publications rose in waves, from 6 in 1992 to 41 in 2021 (Figure 2B). The annual growth rate was 6.85%. The peak of publication appeared in 2013; documents from that year accounted for approximately 5.9% (46/783) of all publications from the period of interest. Although the number of publications decreased after 2013 and reached its lowest point over the last 10 years in 2019, there was a rebound burst in 2020, which indicated that interest in research on HH in the past 2 years grew again and underwent a period of rapid development. To predict the number of publications, a linear regression model was constructed based on the number of annual publications; the formula was as follows:


y=0.9333x+11.634

where x indicates the order of the year. For example, 1992 is the first year in this figure, and $x_{1992}=1$ . The results are also shown in Figure 2B as a dashed purple line. The number of documents published in 2022 was predicted to be 41 (40.57). In addition, Table 1 lists the top 10 most cited publications in HH. Notably, the number of citations was not limited to articles in the HH field. For earlier publications, they may have an advantage in terms of citation counts, which can affect the assessment of their impact. This issue was addressed by using the citation per year index (23). The study by Latronico et al. on central precocious puberty had the highest index (26.83). According to the betweenness centrality calculated by CiteSpace (Supplementary Data Sheet 1), the most important document in this field was a report by Esquenazi et al. of a case treated with MRg-LiTT (24).

**Table 1:** The top 10 most cited publications on HH.

Title	Authors	Years	Sources	Total citations	Citation per year
GLI3 Frameshift Mutations Cause Autosomal Dominant Pallister-Hall Syndrome	Kang S et al.	1997	*Nature Genetics*	366	14.64
Defining the Spectrum of International Practice in Pediatric Epilepsy Surgery Patients	Harvey S et al.	2008	*Epilepsia*	294	21
Neural Correlates of Laughter and Humour	Wild B et al.	2003	*Brain*	244	12.84
Intrinsic Epileptogenesis of Hypothalamic Hamartomas in Gelastic Epilepsy	Kuzniecky R et al.	1997	*Annals of Neurology*	243	9.72
The Role of Subcortical Structures in Human Epilepsy	Norden AD et al.	2002	*Epilepsy & Behavior*	225	11.25
Mirth, Laughter, and Gelastic Seizures	Arroyo S et al.	1993	*Brain*	223	7.69
Mutations Within Sox2/Sox2 are Associated with Abnormalities in the Hypothalamopituitary-Gonadal Axis in Mice and Humans	Kelberman D et al.	2006	*Journal of Clinical Investigation*	223	13.94
Role of the Hypothalamic Hamartoma in the Genesis of Gelastic Fits (A Video-Stereo-EEG Study)	Munari C et al.	1995	*Electroencephalography and Clinical Neurophysiology*	220	8.15
Hypothalamic Hamartomas – with Special Reference to Gelastic Epilepsy and Surgery	Valdueza JM et al.	1994	*Neurosurgery*	201	7.18
Disconnecting Surgical Treatment of Hypothalamic Hamartoma in Children and Adults with Refractory Epilepsy and Proposal of a New Classification	Delalande O et al.	2003	*Neurologia Medico-Chirurgica*	179	9.42

### 3.2. Analysis of journals

The disciplines in which HH is studied can be reflected by the journal types in which documents on HH are published. Through an analysis using the “biblioshiny” website, we obtained the statistical results of the journal (Supplementary Table S2). The top 10 journals that contributed to the publication of HH research are shown in Table 2. The journal with the largest number of publications was ***Epilepsia*** (*n* = 62); it also achieved the highest H-index (*n* = 28), and G-index (*n* = 45) among all journals. Other journals with more than 30 publications included ***Epileptic Disorders*, *Child’s Nervous System*, *Neurosurgery***, and the ***Journal of Neurosurgery***.

**Table 2:** Top 10 journals by number of publications on HH.

Rank	Sources	Publications	H-index	G-index	IF (2021)	JCR (2021)
1	*Epilepsia*	62	28	45	6.740	Q1
2	*Epileptic Disorders*	38	18	27	2.333	Q3
3	*Childs Nervous System*	34	14	24	1.532	Q3
4	*Neurosurgery*	32	12	18	5.315	Q1
5	*Journal of Neurosurgery*	31	20	27	5.526	Q1
6	*Epilepsy & Behavior*	25	12	24	3.337	Q2
7	*Neurology*	23	15	19	12.258	Q1
8	*Journal of Pediatric Endocrinology & Metabolism*	20	7	12	1.520	Q3
9	*Seizure-European Journal of Epilepsy*	19	12	16	3.414	Q2
10	*Epilepsy Research*	17	10	15	2.991	Q2

IF, impact factor; JCR, journal citation reports.

### 3.3. Analysis of authors

Author analysis can assist us in identifying researchers who have made significant contributions to the field of HH research. According to the analysis of authors in bibliometrix, the top 10 authors in terms of the cumulative number of publications were Kerrigan JF, Ng YT, Rekate HL, Regis J, Kameyama S, Harvey AS, Masuda H, Biesecker LG, Rosenfeld JV, and Bartolomei F (Table 3 and Supplementary Table S3).

**Table 3:** Top 10 most productive authors in HH research.

Rank	Authors	Publications	H-index	G-index	Local citations
1	Kerrigan JF	50	22	33	801
2	Ng YT	31	17	27	504
3	Rekate HL	26	17	25	520
4	Regis J	19	11	17	287
5	Kameyama S	17	10	15	248
6	Harvey AS	16	13	16	512
7	Masuda H	16	9	14	193
8	Biesecker LG	15	11	15	220
9	Rosenfeld JV	15	10	14	488
10	Bartolomei F	14	9	12	255

**Figure 3:**
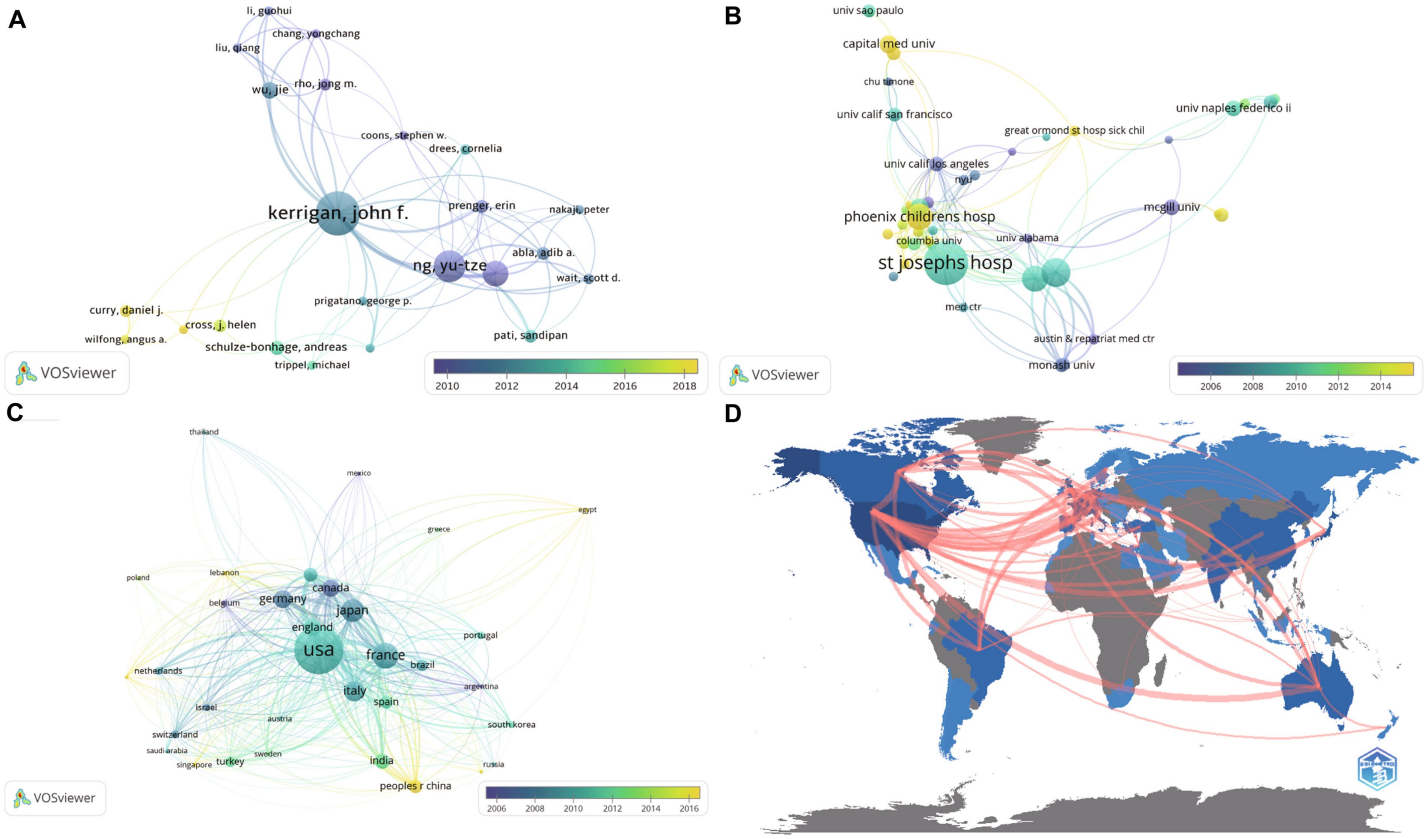
Authorship analysis, institutional analysis, and country analysis. Figures **(A)**–**(C)** were performed in VOSviewer based on co-authorship analysis. Each node represents an item, including author **(A)**, institution **(B)**, and country **(C)**. The size of the node represents the number of published documents. The interconnecting line between the nodes represents the cooperative relationship between different items. The thickness of the line indicates the relatedness of items based on the number of co-authored documents. The color of the node indicates the year in which the literature was published. The bluer the nodes, the earlier the documents were published; the yellower the nodes, the later the documents were published. **(D)** International collaborations analyzed by biblioshiny.

Research and publication are inseparable from the close collaboration of scholars of the highest caliber. In VOSviewer, co-authorship analysis is based on the analysis of documents published by co-authors, which can show the degree of close cooperation between authors (Figure 3A). The color of the node indicates the year in which the literature was published. The bluer the node, the earlier the documents were published; the yellower the node, the later the documents were published. It can be seen from the figure that Kerrigan (Kerrigan JF) and Ng (Ng YT) are important collaborators in HH research. They are in the important position of the HH research, as they published numerous literature and have close connections with many other researchers. Furthermore, the authorship analysis revealed many influential new scholars (yellow nodes), such as Gaillard, William D., Tisdall, Martin, Curry, Daniel J., Ali, Irfan, and Wilfong, Angus A.

### 3.4. Analysis of research institutions

Through institution analysis, it was possible to identify the world’s leading scientific research institutions and hospitals in the field of HH, which can aid patients in obtaining effective treatment. In our analysis of research institutions, we found that the Barrow Neurological Institute has 148 published papers, followed by University of Melbourne (51 publications, Australia), All India Institute of Medical Science (45 publications, India), Royal Children’s Hospital (34 publications, Australia), Capital Medical University (31 publications, China), Aix-Marseille University (21 publications, France), McGill University (21 publications, Canada), University of Naples Federico II (18 publications, Italy), Alfred Hospital (17 publications, Australia), and Baylor College of Medicine (17 publications, United States) (Supplementary Table S4).

Comparable results were obtained in the VOSviewer analysis (Figure 3B). The institutional analysis based on co-authorship demonstrated that Barrow Neurological Institute at St. Joseph’s Hospital was in the leading position in HH research. That institute conducted extensive research on numerous aspects of this disease. Phoenix Children’s Hospital and Capital Medical University have also been important forces in the study of HH in recent years.

### 3.5. Analysis of countries or regions

There are substantial disparities in science and health care among nations and regions. In terms of the number of publications grouped by country, the United States has a major advantage and leads the globe in HH research, with 240 publications and 5,351 citations. It was followed by France (89 publications, 2,275 citations), Japan (74 publications, 1,184 citations), Italy (64 publications, 1,610 citations), Germany (53 publications, 1,444 citations), Canada (48 publications, 1,459 citations), England (46 publications, 1,313 citations), India (41 publications, 267 citations), Australia (35 publications, 1,181 citations), and P.R. China (34 publications, 175 citations). The numbers of citations and publications were not exactly proportional.

The visualized results revealed more profound information (Figure 3C). The size of each node represents the number of publications. The color ranges from dark blue to light yellow, indicating the order of studies from past to present. Experts in Belgium and Canada performed much earlier work in the HH field (blue color). Although China is only in 10th place in number of publications, its participation in this field of research is increasing (yellow color). This feature can also be seen in Egypt and Lebanon. Figure 3D shows the close international exchanges and cooperation in the field of HH research.

### 3.6. Analysis of research hotspots

**Figure 4:**
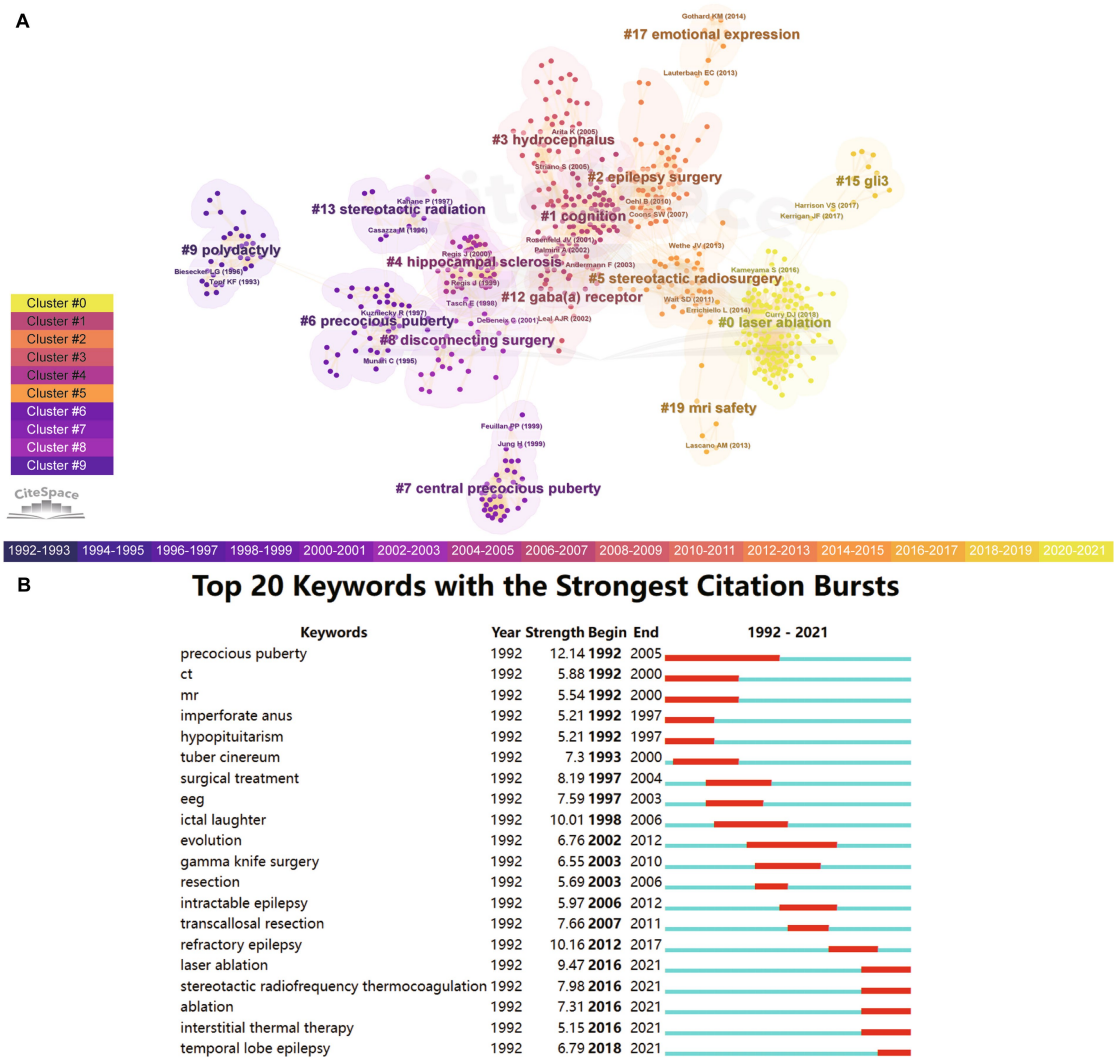
Research hotspots. **(A)** Cluster analysis based on keywords. Each node represents a document. The more purple the nodes, the earlier the documents were published; the yellower the nodes, the later the documents were published. Studies with similar themes were clustered together. Each cluster represents a specific medical concept. **(B)** Citation burst analysis. It demonstrates the strength and duration of citation for different key words.

Analyzing the historical evolution of research hotspots is a crucial application of bibliometrics, helping to determine the appropriate direction of future research. CiteSpace was used to detect the research hotspots in the field of HH. Studies with similar themes were clustered together in the hierarchical cluster analysis. Figure 4A demonstrates the overall evolution of manuscript clusters related to HH over time. The more purple the nodes, the earlier the documents were published; the yellower the nodes, the later the documents were published. The earliest research cluster, shown on the left side of the figure, focused on polydactyly. Topf (1993) and Biesecker (1996) were key authors in this cluster. In the middle of the figure, the number of clusters increased, ranging from symptomatology (precocious puberty, hippocampal sclerosis, and hydrocephalus) to therapeutic methods (disconnecting surgery, epilepsy surgery, and stereotactic radiosurgery). With the advent of precise and minimally invasive techniques in neurosurgery, the availability and interest in HH treatment has grown steadily over time. Laser ablation has been one of the hotspots of HH research in recent years. In basic research, there has been considerable attention to the transcription factor GLI3, which is part of the Hedgehog (Hh) signaling pathway. This transcription factor was associated with genetic disease, including HH, Greig cephalopolysyndactyly (GCPS) (25), PHS (26), and polydactyly (27) as well as cancer and disorders of organ development (25).

Through citation burst analysis, we found that multiple keywords play an important role in the historical development of HH studies (Figure 4B). Early studies mainly focused on symptomatology keywords such as precocious puberty (1992–2005), imperforate anus (1992–1997), ictal laughter (1998–2006), intractable epilepsy (2006–2012), temporal lobe epilepsy (2018–2021), diagnostic measures such as CT (1992–2000), MRI (1992–2000), and EEG (1997–2003). At the same time, we can intuitively see the evolution of the popularity of different treatment measures in the figure. Surgical treatment was used in the early stages (1997–2004), followed by Gamma Knife surgery (2003–2010), transcallosal resection (2007–2011), laser ablation (2016–2021), stereotactic radiofrequency thermocoagulation (2016–2021), ablation (2016–2021), interstitial thermal therapy (2016–2021), etc. Analysis on biblioshiny showed similar results (Supplementary Figure S1). This succession of treatment measures reflects the innovation in the surgical treatment of HH.

## 4. Discussion

Located at the ventral hypothalamus, HH is a rare benign lesion (7). Based on the bibliometric analysis, we provide an in-depth review of developments in HH diagnosis and treatment over the past 30 years. Through in-depth analysis of the documents, as well as publication by authors, institutions, and countries, we can observe the historical development of HH research and the continuous improvement of treatment strategies.

In our research, the annual number of publications in the field of HH showed a fluctuating upward trend. Thus, with the development of neurosurgical techniques, HH has attracted increasing attention from researchers in recent years.

According to the analysis of journals, neurology, neurosurgery, and epilepsy are the primary journal categories that have addressed the topic of HH. Among them, the ***Journal of Neurosurgery*** ranked only fifth in the number of publications. However, its H-index was 20, ranking second. In 2003, the number of publications in ***Epileptic Disorders*** in the field of HH increased suddenly, with 16 publications in that year. The cumulative number of publications ranked first in the field of HH since then. However, since 2011, ***Epilepsia*** has led the way in HH based on its relatively steady number of annual publications in this field.

In terms of the authors analysis, Kerrigan JF had the most papers published in the field of HH. In addition, Kerrigan JF was the author with the highest number of local citations, which refers to the total citations cited by all publications in the same field (in this case, HH research). The number of local citations indicates the importance of an author’s research in a field. Kerrigan and Ng play a crucial role in the research on HH as collaborators. They published a comprehensive review in HH in 2005, noting that HH was an essential model for secondary and subcortical epilepsy. During that time, many researchers believed that a surgical approach from the superior aspect was the most effective treatment for a majority of hypothalamic lesions, which led to the popularity of surgical treatment for HH (28). Advances in the field of HH are also dependent on the continued emergence of new scholars. For example, Curry and Gaillard et al. proposed that preoperative resting-state functional MRI (rs-fMRI) contributed to improving the outcome of epilepsy surgery (29). Through a case series study, Wilfong et al. also found that MRI-guided stereotactic laser ablation (SLA) was a novel, safe, valuable, and effective treatment modality for epileptogenic HH (30). Curry, Raskin, Ali, and Wilfong collaboratively reported 71 HH patients operated on with laser ablation, which was demonstrated to be safe and effective (31). Tisdall et al. presented 46 pediatric cases to examine psychiatric comorbidities in HH, including externalizing and internalizing disorders. The former symptoms were associated with epilepsy and male sex. However, there were no clear pathogenic factors correlating with internalizing disorders and precocious puberty (5).

The institution analysis demonstrated that the Barrow Neurological Institute at St. Joseph’s Hospital had a significant advantage over other institutions (Supplementary Table S5), and has published nearly three times as many papers as the second-place institution. In 2011, St. Joseph’s Hospital researchers studied sodium homeostasis post-surgery in HH patients and found DDAVP to be effective for salt replacement therapy (9). Two years later, researchers in this institution found that HH patients could achieve mild to moderate improvements in intelligence if they did not have surgical complications (8). Several American institutions occupy the top 10 positions, dominating the rankings. It should be noted that Capital Medical University, as a representative of China’s medical institutions, ranked fifth, indicating that it has a certain influence on HH research.

We obtained the similar result in the analysis of nations and regions. United States occupied a pivotal position in the field of HH. It was followed by European countries. Notably, a catch-up development in HH research was uncovered in some developing countries, such as India, China, Egypt, and Lebanon.

The most important role of bibliometrics analysis is to assist in identifying historical evolution, research hotspots and future research directions. The early literature referring to HH mainly focused on PHS, which was first described in 1980. Patients with this genetic disease commonly present with HH, central polydactyly, and other malformations (32). The causative gene of PHS is GLI3, which is located on chromosome 7p13 with an autosomal dominant inheritance pattern (33). Biesecker et al. further analyzed PHS and concluded that HH is a clinical manifestation that is not exclusive to PHS (34). Topf et al. also reported a child patient and his father who were diagnosed with PHS (35) (Figure 4A). A later study found that PHS patients had a lower incidence of epilepsy than patients with isolated HH, and all PHS patients with seizures had hamartomas. The endocrine symptoms in patients with isolated HH were also more severe and difficult to control than those of PHS (36). This suggests that isolated HH should be taken seriously. Precocious puberty is a very important endocrinal manifestation and a crucial keyword in the literature on HH. Early studies showed that HH acted on the hypothalamic–pituitary–gonadal axis through the production and release of luteinizing hormone-releasing factor (37). Later, a study used a long-acting gonadotropin-releasing hormone (GnRH) analog to treat gelastic seizures and precocious puberty in HH and achieved a certain degree of efficacy (38). Another study found that GnRH analogs increased the mean ovarian volume (MOV) in HH and idiopathic precocious puberty (IPP) patients, but did not affect incidence of oligomenorrhea, obesity or neurological disorders (39). Surgical excision is rarely used to treat precocious puberty unless accompanied by refractory epilepsy (7). The word cloud analysis of the keywords yielded similar results (Supplementary Figure S2).

Conventional antiepileptic drugs, such as carbamazepine and lamotrigine, had no significant therapeutic effect on HH. These drugs may not penetrate this lesion well and therefore respond less well to this particular type of epilepsy. The goal of treatment is to minimize the transformation and progression of the seizure type, not to reduce the frequency of the gelastic seizure itself (6). In many subsequent clinical studies, the drug resistance rate in HH was at least 50% (40).

The pathogenic mechanism by which HH causes epilepsy is poorly understood. Anatomically, HH is closely related to the immediate vicinity of the mammillary bodies. This indicates that connections to the limbic system may be the underlying epileptogenic mechanism (41). Laughter in the form of gelastic seizures is a special and unique manifestation of this disease. Studies have shown that there are two potential neuronal pathways for laughter. The first is the involuntary (emotionally driven) system, including the thalamic/hypo-and subthalamic areas, the amygdala, and the dorsal/tegmental brainstem. The second is the voluntary system, which includes premotor/frontal opercular areas and leads through the motor cortex and pyramidal tract to the ventral brainstem. However, giggling in HH cannot be attributed to the former simply because of its anatomic proximity to the hypothalamic areas, nor can it be attributed to the latter merely because the laughter is not accompanied by emotional fluctuation (42). In fact, HH epilepsy may also be associated with the mesial temporal lobe (1). A previous study also demonstrated that gelastic seizures originating in the temporal lobe were associated with emotion, whereas those in the HH were not (42). Lu et al. proposed that the dentato-rubro-thalamic tract and cerebro-ponto-cerebellar tract might be involved in gelastic seizures in patients with HH by using ^18^F-fluorodeoxyglucose-positron emission tomography (^18^F-FDG-PET) (17). These findings likely highlight why HH-related epilepsy is difficult to treat.

Usually, the diagnosis of HH is difficult due to the deep location and small size of the lesion. There may be false negative EEG results, as 75% of patients with gelastic seizures had no ictal EEG change in a previous study (43). Through bibliometric analysis, we can see that in early studies, essential keywords included CT and MRI (Figure 4B). This indicates the development of imaging techniques in HH. As early as 1987, CT examinations were performed to detect cerebral diseases such as HH that cause precocious puberty in children (44). The MRI diagnosis of HH also appeared in 1988 (45). MRI is more sensitive than CT in identifying hamartomas of small size (46). In recent years, SEEG has become a more accurate diagnostic method.

Traditional surgical treatments include transcallosal, interforniceal, and transventricular resection of the HH with frameless stereotactic navigation (47). Surgical resection can be the most thorough treatment when the epileptogenic foci are precisely identified (48). In the absence of surgical complications, patients with tumor resection may experience mild to moderate increases in intelligence after surgery (8). In addition, younger patients and those with short courses of seizures fared better after surgery. In one study, the immediate postoperative complication rate was 13.5%, and complications included short-term memory deficits, complete hemiplegia, transient facial palsy, postoperative infections, diabetes insipidus, and immediate status epilepticus. Some patients also suffered from permanent complications, including motor deficits, weight gain, and long-term memory deficits (49).

In recent years, with the development of minimally invasive techniques and robotic surgery (12), stereotactic radiosurgery (SRS) has become an important therapy modality for HH with minimal tissue trauma, including Gamma Knife and CyberKnife (12). Sufficient irradiation to the margin of the HH can obtain great seizure remission rates (50). One study showed that 39.6% of patients achieved complete seizure cessation (Engel class I) after Gamma Knife treatment. A total of 29.2% of patients were almost seizure free with rare, disabling seizures (Engel class II). More than half of the patients who were aggressive before surgery experienced remission of their psychiatric symptoms (51). However, the impact of this treatment is not immediate. Seizures may even worsen in the initial stages of treatment due to the degree of tissue inflammation and edema. In a recent study, 80% of patients who received CyberKnife therapy achieved a good clinical outcome (Engel classes I and II). However, the improvement appeared only after 6 months of postoperative follow-up (52).

Recently, thermocoagulation has also emerged as a promising technique. With stereotactic guidance, thermocoagulation technology can cause a sharp local temperature increase at the site of the lesion. It denatures proteins in brain tissue at temperatures above 45°C. Thermocoagulation works faster than SRS therapy and causes less damage to surrounding normal tissue. At present, two of the main approaches are stereotactic RF-TC and MRg-LiTT.

RF-TC can be guided by MRI or SEEG. The aim of this procedure is to use heat generated by electric currents to separate HH from normal hypothalamus tissue as much as possible. According to Kameyama et al., the overall seizure freedom rate was 71%. Approximately 30% of patients undergo more than one thermocoagulation procedure (53). In SEEG-guided RF-TC, patients are first implanted with several SEEG electrodes. Then, patients undergo long-term video intracranial EEG monitoring to identify the epileptogenic foci. SEEG electrodes are then used for thermocoagulation based on the electrical current between contact points. Researchers from Xuanwu Hospital of Capital Medical University conducted an in-depth study in this area. The study of Wei et al. demonstrated that all patients achieved favorable prognoses (Engel classes I and II) after SEEG-guided RF-TC (16). For patients with mesial temporal lobe epilepsy associated with hippocampal sclerosis (MTLE-HS), Fan et al. further improved the effect of thermocoagulation through the three-dimensional cross-bonding of various electrode contacts, which led to better clinical outcomes (54). Further research by Dai et al. showed that these novel techniques also achieved good results in insular epilepsy (55). In fact, MRI and EEG were not completely independent, and both techniques were used in these two types of therapy, although the degree of participation varied.

In this study, we found that laser ablation, also designated LiTT, has become a major research hotspot in recent years. MRg-LiTT has become an important treatment strategy for drug-resistant epilepsy (14, 56). The intensity of the laser energy and the duration of treatment are the two core components of therapy. With the help of the automatic closed-loop control of the LiTT software and intraoperative real-time MRI, neurosurgeons can carry out the surgery more efficaciously, safely, and easily. LiTT has been expected to become the first-line treatment for seizures caused by HH (57). DI Perna et al. reported that nonrobotic frameless MRg-LiTT was an effective method for treating HH (15). Curry et al. reported a series of 71 patients with HH who were operated on with laser ablation. More than 90% of those patients were free of gelastic seizures after 1 year. This demonstrated the excellent effect of laser ablation (31). Currently, a multicenter clinical trial on MRg-LiTT for medical refractory epilepsy is underway in Capital Medical University (NCT04569071). We hope that this clinical trial will help to popularize the MRg-LiTT as a safe and effective treatment to medical refractory epilepsy, including HH.

Through bibliometric analysis, we suggest that the focus of future research in the field of HH will be on the design of treatment strategies primarily based on MRg-LiTT and RF-TC, which are currently considered research gaps. Specifically, determining the laser irradiation temperature and time for MRg-LiTT treatment for different sizes of HH will require more accurate methods. The position of SEEG electrodes can be optimized to achieve better outcomes for RF-TC guided procedures. Three-dimensional cross-bonding techniques may have potential in the treatment and prognosis of HH. It is important to minimize damage to surrounding normal tissue caused by various minimally invasive techniques. Additionally, physiological studies of laughter may drive the development of new drugs to better treat and control gelastic seizures caused by HH.

The present study included several limitations. First, this study only included literature from the SCI-EXPANDED database. Literature that was not in this database was not included in the study. Besides, the literature included in this study was mainly in English. These issues may still require improvement in bibliometric analysis methods and software.

## 5. Conclusion

This bibliometric study was the first to assess HH-related literature worldwide. It provides clinicians and scientists with new insights into HH and the development of knowledge on this topic. The present study offers new ideas and insights for the diagnosis and treatment of HH and other epilepsy diseases manifested by gelastic seizures. Clarifying the pathogenesis of HH will still depend on the integration of neuroscience, clinical medicine, and other research disciplines. The minimally invasive techniques represented by MRg-LiTT and RF-TC provide a new and safer therapeutic strategy for HH treatment and have broad research prospects.

## Data availability statement

The original contributions presented in the study are included in the article/Supplementary material, further inquiries can be directed to the corresponding authors.

## Author contributions

GZ conceived and designed the study. DL performed the bibliometrics analysis and drafted the manuscript. TW collected the data and performed the bibliometrics analysis. YY provided the methodology and reviewed the results. XF and SC reviewed and revised the draft manuscript. PW carried out the quality control and finalized the manuscript. GZ and YS provided expert consultations and finalized the manuscript. All authors contributed to the article and approved the submitted version.

## Funding

This study was supported by the National Natural Science Foundation of China (82030037, 81801288, 81871009), STI2030-Major Projects (2021ZD0201801), and the Translational and Application Project of Brain-inspired and Network Neuroscience on Brain Disorders, Beijing Municipal Health Commission (11000022T000000444685).

## Conflict of interest

The authors declare that the research was conducted in the absence of any commercial or financial relationships that could be construed as a potential conflict of interest.

## Publisher’s note

All claims expressed in this article are solely those of the authors and do not necessarily represent those of their affiliated organizations, or those of the publisher, the editors and the reviewers. Any product that may be evaluated in this article, or claim that may be made by its manufacturer, is not guaranteed or endorsed by the publisher.

## Footnotes

^1^https://www.nlm.nih.gov/mesh/
