# Supplementary figures and images for: Advances in hypothalamic hamartoma research over the past 30 years (1992–2021): a bibliometric analysis
Source: Front Neurol. 2023 Jun 6;14:1176459. doi: 10.3389/fneur.2023.1176459 (PMC10322195; doi:10.3389/fneur.2023.1176459)

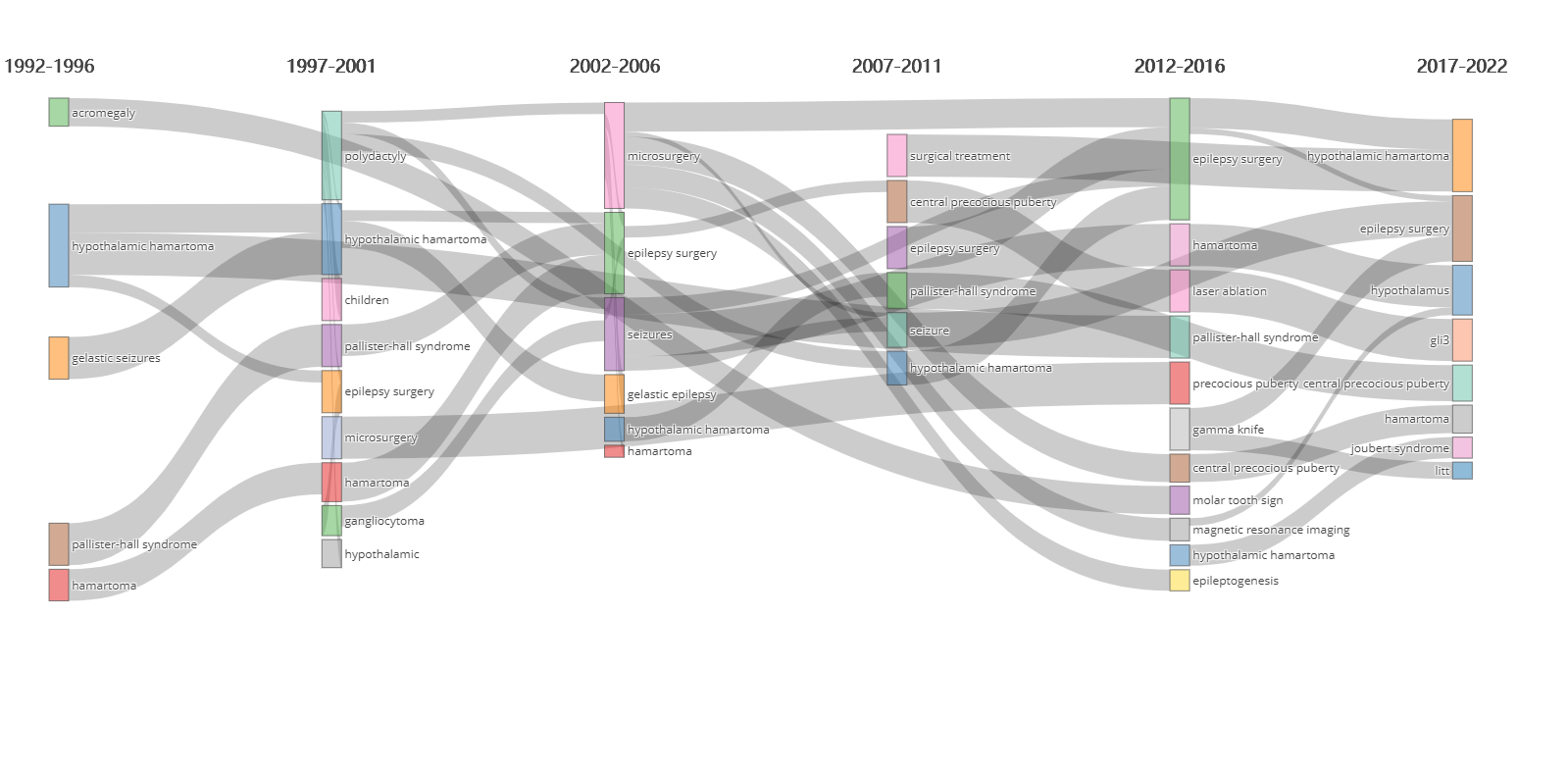

Supplement: Supplementary file 6 [file Image_1.PNG]

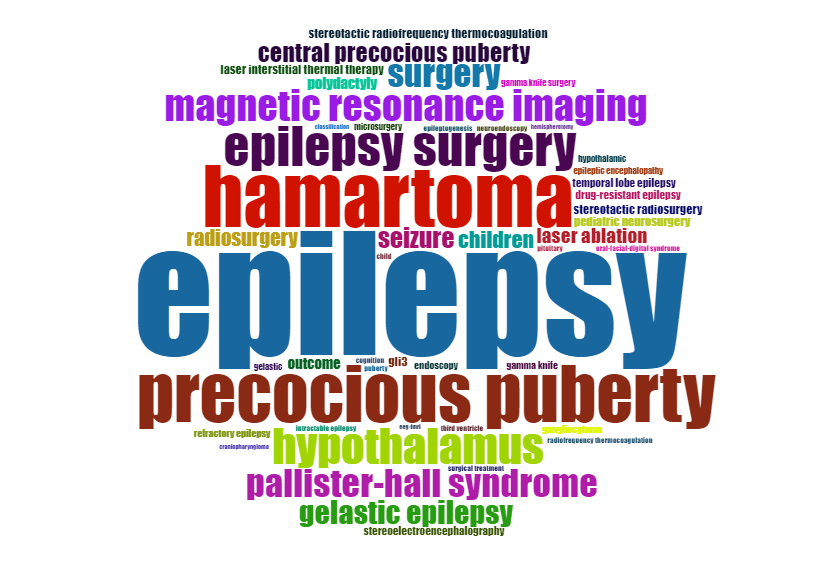

Supplement: Supplementary file 7 [file Image_2.PNG]
